# Generation of Lung Adenocarcinoma DNA Aptamers for Cancer Studies

**DOI:** 10.1371/journal.pone.0046222

**Published:** 2012-10-17

**Authors:** Elizabeth Jiménez, Kwame Sefah, Dalia López-Colón, Dimitri Van Simaeys, Hui William Chen, Melvyn S. Tockman, Weihong Tan

**Affiliations:** 1 Department of Chemistry, University of Florida, Gainesville, Florida, United States of America; 2 Department of Chemistry, University of North Carolina, Chapel Hill, North Carolina, United States of America; 3 Department of Oncology Sciences, Moffitt Cancer Center, Tampa, Florida, United States of America; Barts & The London School of Medicine and Dentistry, Queen Mary University of London, United Kingdom

## Abstract

Lung cancer is the most lethal malignancy in the world, and each year thousands of people die from this disease. Early detection has proven to increase the 5-year survival for this cancer in general, independent of the origination site in the lung. To address this challenge, we have used cell-based SELEX (**Systematic Evolution of Ligands by Exponential Enrichment**) to select a panel of aptamers capable of distinguishing lung adenocarcinoma cells from normal lung epithelial cells. These aptamers bind at physiological and formalin-fixed conditions and display affinity for their targets with apparent K_d'_s in the nanomolar range. Our findings suggest that the selected aptamers have the potential to be used in clinical settings, as well as to improve classification of nonsurgical specimens, another current challenge in lung cancer.

## Introduction

Lung cancer is the second most common type of cancer and the leading cause of cancer-related deaths worldwide [1]. In the United States, it is estimated that 226,160 new cases and 160,340 deaths will occur in 2012 (for Non-small cell and small cell combined). Compared to other cancer types, lung neoplasms are highly heterogeneous, with tumors displaying more than one subtype as a common feature [2]. The vast majority of lung neoplasms are carcinomas, which are generally classified as either non-small cell lung carcinomas (NSCLC) or small cell lung carcinomas (SCLC) on the basis of morphological analysis by stained histological samples [3]–[4]. NSCLC is the most common lung cancer type, comprising 85% of all lung cancer cases; yet it is a more passive cancer type. NSCLC is composed of three different subtypes: adenocarcinoma (ADC), squamous cell carcinoma (SCC), and large cell carcinoma (LCL). On the other hand, SCLC is less common, comprising 15% of all lung cancer cases, but it is more aggressive. Smoking is a risk factor heavily associated with lung cancer, specifically SCC [5]–[10]. Lung adenocarcinoma is commonly developed by patients who have never smoked, and genetic changes are often associated with its onset.

Since most people with lung cancer at the early stage do not display symptoms, more than 70% of lung cancer cases are diagnosed at later stages, for which the 5-year survival rate is small. Therefore, research aimed at early detection, which is critical to reducing mortality and morbidity, has turned to the development of suitable aptamers. Aptamers are short, single-stranded DNA or RNA oligonucleotides which are highly specific target recognition elements based on their unique three-dimensional shapes [11]–[12]. While the process known as SELEX (Systematic Evolution of Ligands by Exponential Enrichment) was originally used to select aptamers against targets such as purified proteins [13]–[14], cell-based SELEX has become the newest method of selecting aptamers against whole cells, especially those aptamers targeting surface proteins overexpressed in cancer cells.

Among their many advantages, aptamers have shown no, or extremely low, immunogenicity, permitting *in vivo* studies using these probes [15]–[18]. They have also been popularized as alternatives to antibodies, because of aptamers' low cost (no animals necessary for production), easy chemical modification, and cellular uptake capability. In addition, because aptamers are small in length with generally 15 to 100 nucleotides (nt), they have better tissue penetration compared to antibodies. In 2004, Macugen, an anti-VEGF (Vascular Endothelial Growth Factor) inhibitor, became the first aptamer approved by the Food and Drug Administration (FDA) for Age-Related Macular Degeneration (AMD) [19]. Other aptamers remain in clinical trials [20], and have demonstrated great potential in the biomedical field, including separation, drug delivery and target-probe measurement. This report describes the use of cell-SELEX to select a panel of aptamers capable of distinguishing between lung adenocarcinoma and normal lung epithelial cells.

## Results and Discussion

**Figure 1:**
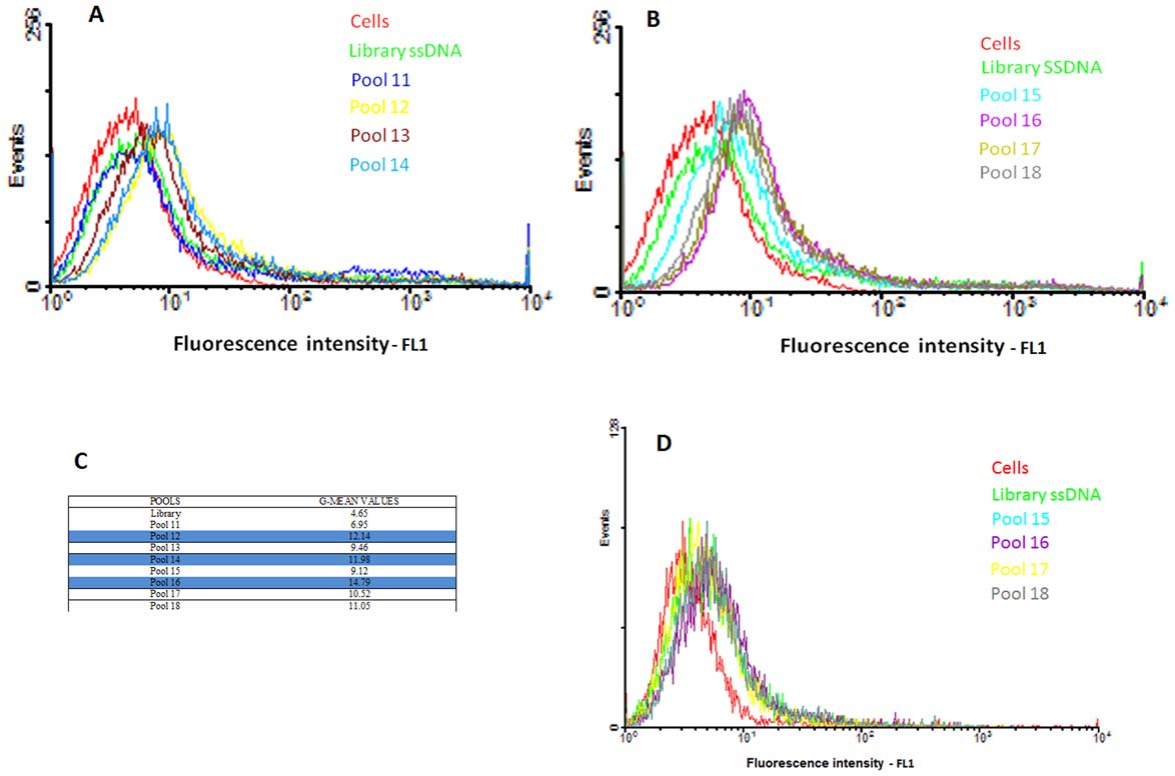
Progress of cell-SELEX methodology. Binding assay of pools 11–18 with H23 (A–B) and pools 15–18 with HBE135 E6/E7 (C). Flow cytometry assay to monitor the binding of selected pool with H23 cells (target cells) and HBE135 E6/E7 (negative cells). The green curve represents the background binding of unselected DNA library. For H23 cells, there was an increase in binding capacity of the pools as the selection progressed, whereas there was little change for the control cells, HBE 135 E6/E7. **Aim figure 1**: The aim of this figure is to show how the cell-selection process was enriched for H23 cell line but not for HBE135E6/E7 cell line.

Since their discovery, aptamers have been generated against different targets, including proteins, peptides, and living cells [21]–[22]. To isolate aptamers capable of differentiating lung adenocarcinoma cells from normal lung epithelial cells, we used the cell-based SELEX strategy. H23 lung adenocarcinoma and HBE 135-E6/E7 normal epithelial lung were used as positive and negative cell lines, respectively. An initial ssDNA random library containing approximately 10^14^ different sequences of 80 nucleotides (nt) was enriched by sequential binding with the target cells, elution and subsequent amplification by PCR for 18 rounds. These DNA sequences could recognize H23 cell-surface membrane proteins which are potential markers for targeted therapy. In earlier rounds of the process, counter selection was introduced in order to remove possible sequences binding common proteins on both target and negative cell lines. This procedure was performed every other round throughout the selection. Sequences binding to target cells were eluted and PCR-amplified, after which ssDNA was recovered and used to monitor the selection process by flow cytometry. Because the ssDNA pools were enriched with sequences specific for the target, an increase in fluorescence intensity was first noticed in round 12 (Figure 1A), indicating that those sequences showed better binding on the surface of H23 cells compared to the initial library. As the selection progressed, the fluorescence intensity of the subsequent pools gradually increased until a steady state in fluorescence intensity was observed in rounds 17 and 18 (Figure 1B), indicating that maximum binding had been achieved. In contrast, such enrichment was not observed when the enriched pools were tested against normal lung epithelial cells (Figure 1D), implying that sequences contained in those pools were capable of distinguishing lung adenocarcinoma cells from normal lung epithelial cells. Therefore, pools 14, 16 and 18 were selected to be sequenced as aptamer candidates (Figure 1C).

**Figure 2:**
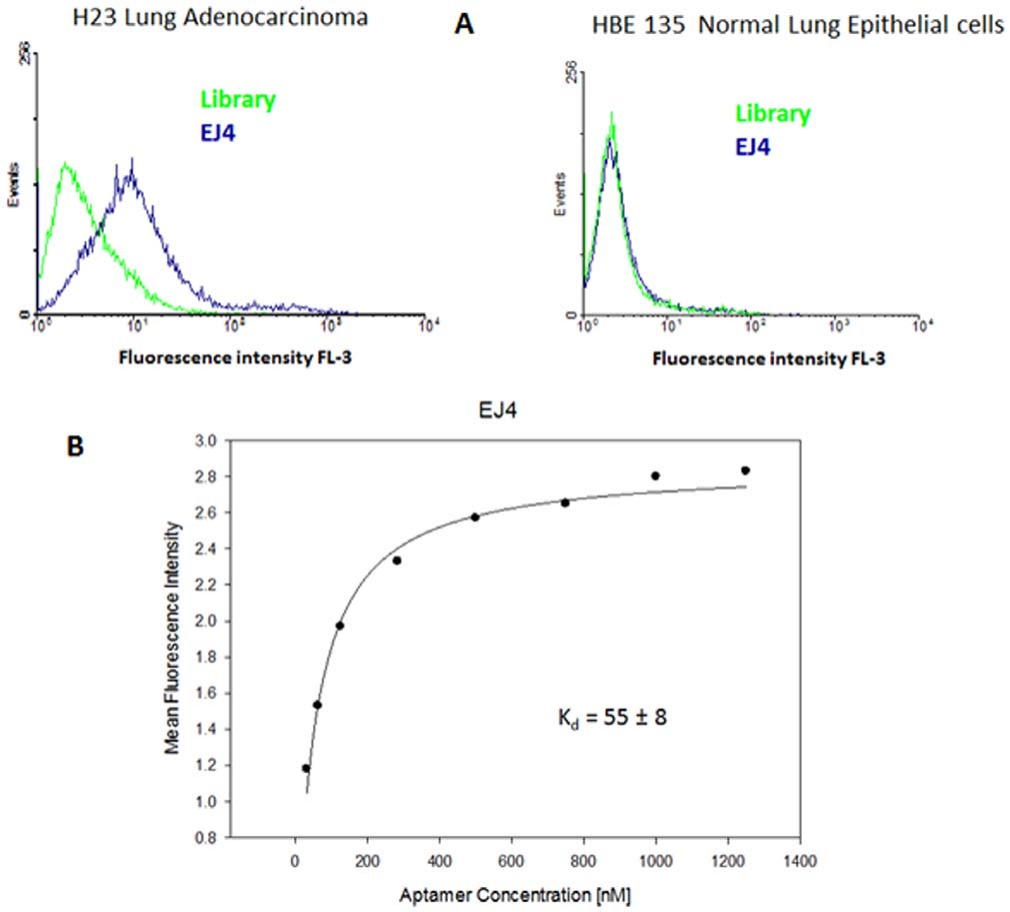
Characterization of selected aptamers. Flow cytometry assay for the binding of the selected aptamer EJ4 with H23 (target cell line) and HBE135 E6/E7 (negative cell line). The green curve represents the background binding of a random sequence (library). **Aim Figure 2**: The aim of this figure is to show that the candidate aptamers are indeed aptamers because they do bind with the positive cell line (H23), but do not bind with the negative cell line (HBE135E6/E7).

The sequencing process began with the construction of the 454 sequencing library by PCR-addition of 454-specific primers to the 3′- and 5′-ends of the enriched pools. To identify the pool for each sequence, a unique identification code (middle identification code, MID) was also PCR-introduced to the 454 sequencing library. The insertion of primers in the enriched pools was confirmed by gel electrophoresis (data not shown). After 454 sequencing at the UF ICBR core, around 7,000 sequences were retrieved and analyzed. They were grouped on the basis of the MID corresponding to the same pool, and primers were removed by a Perl program. The sequences containing only the random region were then aligned using the online program MAFTT 6.0 [23], and six aptamer families corresponding to the most abundant sequences were chosen as aptamer candidates. They were chemically synthesized, biotin-labeled at the 3′-end, purified by HPLC, and quantified. All aptamers displayed binding with H23 lung adenocarcinoma cells, while no significant binding was observed with the control cell line HBE 135 E6/E7, normal epithelial lung cells (figure 2 and figure S1). These results indicate that successful negative selection had been carried out and that selected aptamers could distinguish between lung adenocarcinoma and normal lung epithelial cells, an outcome which confers these aptamers with the potential for use in lung cancer diagnosis, monitoring tumor progression and treatment, or other relevant applications.

All the selected aptamers showed binding affinities (apparent K_d_'s) to the H23 cell line in the nanomolar range (45–250 nM) (Table 1 and figure S6). These results suggest that these aptamers will be widely applicable, as they tightly bind to their target. Further studies were carried out to characterize the selected aptamers. The binding was further tested against lung cancer cell lines, as well as other cell lines, including ovary and colon, as shown in Table 2. Aptamers EJ2, EJ4 and ADE1 displayed some recognition towards one or more of the following cell lines: TOV21G, DLD1, and H460. In the case of DLD1, a colon adenocarcinoma cell line, it was interesting to see some recognition by the selected aptamers, suggesting that a possible common cell-surface target is present in these two cell lines, since they belong to the same histological group. In a previous study in our lab, the target protein of Sgc8 aptamer was demonstrated to be PTK7 [24], a pseudo-kinase protein present in CEM cells (the target cell line for that selection), as well as other cancers, including ovary, lung, colon, breast and some leukemia cell lines, indicating that common proteins can be present as a result of cancer [25]–[26]. Aptamers, EJ4 and ADE1 also showed particular specificity towards the lung adenocarcinoma cell line with significant increase in fluorescence intensity with respect to a random sequence, but not to normal lung cells. These results suggest that these aptamers could be used for lung cancer studies.

**Table 1:** Aptamer sequences and their dissociation constant (Kd_s_) aptamers and pool %.

Name	Sequences	Kd (nM)	% pool
**ADE1**	5′AGT GGT CGA ACT ACA CAT CCT TGA ACT GCG GAA TTA TCT AC 3′	70±5	4,7
**ADE2**	5′ GAG CCC TAT CTC ACA CCG CAC CCG CAA ACT ATC ATC CTACAT G 3′	208±38	0,53
**EJ2**	5′ AGT GGT CGA ACT ACA CAT CCT TGA ACT GCG GAA TTA TCT AC 3′	45±5	7,3
**EJ4**	**5′** GAA GAC GAG CGG CGA GTG TTA TTA CGC TTG GAA ACA ACC CC 3′	60±8	10,2
**EJ5**	5′ TAC GGG CTG GAT CCA CTG TTA CGG CGT GTA TCC GCT ATC AA 3′	122±11	0,71
**EJ7**	**5′** GAA GAC GAG CGG CGA GTG TTA TTA CGC TTG GAA ACA ACC CC 3′	55±8	5,8

**Table 2:** Binding of Adenocarcinoma aptamers with other cancer cell lines.

Cell line	Name	ADE1	ADE2	EJ2	EJ4	EJ5	EJ7
**H23**	**Lung Adenocarcinoma**	+++	+++	++++	++++	++	+++
**HBE 135**	**Normal lung bronchial cell**	_	_	_	_	_	_
**A549**	**Lung Adenocarcinoma**	++	_	_	++	_	_
**H460**	**Large cell carcinoma**	_	_	++	++	_	_
**H520**	**Squamous cell carcinoma**	_	_	_	_	_	_
**CAOV3**	**Ovary adenocarcinoma**	_	_	_	_	_	_
**TOV21G**	**Ovary clear cell carcinoma**	++	_	++	++	_	_
**DLD1**	**Colon adenocarcinoma**	_	_	+++	_	_	_

A threshold based on the fluorescence intensity of FITC in the flow cytometric analysis was chosen so that 99% of cells incubated with the FITC-labeled unselected DNA library would have lower fluorescence intensity than the threshold. When the FITC-labeled aptamer was allowed to interact with the cells, the percentage of the cells with fluorescence above the set threshold was used to evaluate the binding capacity of the aptamer to the cells. (−) 0<10%, 10–35% +, 35–60% ++, 60–85% +++, >85% ++++.

**Figure 3:**
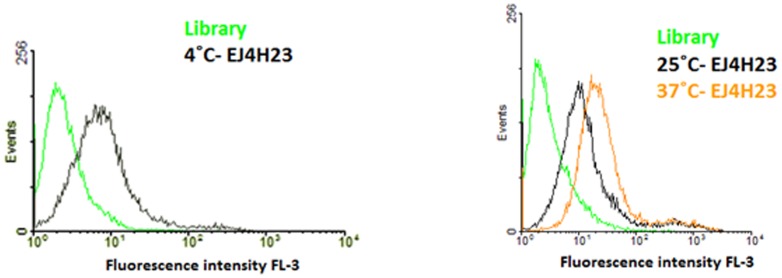
Aptamer binding at physiological conditions. Flow cytometry assay for selected aptamer EJ4 with H23 (target cell line) at physiological temperature (37°C). Binding at 4°C was used as the positive control. The green curve represents the background binding of a random sequence (library). **Aim Figure 3**: The aim of this figure is to demonstrate the binding of the selected aptamer EJ4 at 25°C and physiological temperature, 37°C.

Flexible binding of aptamers at different temperatures can expand their repertoire of applications. Since the selection was performed at 4°C, we performed binding assays at 25°C and 37°C (Figure 3 and Figure S2). As shown in Figure 3 and Figure S3, aptamers ADE1, ADE2, EJ2, EJ5 and EJ7 conserved binding at 25°C and 37°C with fluorescence intensities similar to those at 4°C. This is particularly important in assays carried out under physiological conditions, such as those assessing *in vivo* applications.

**Figure 4:**
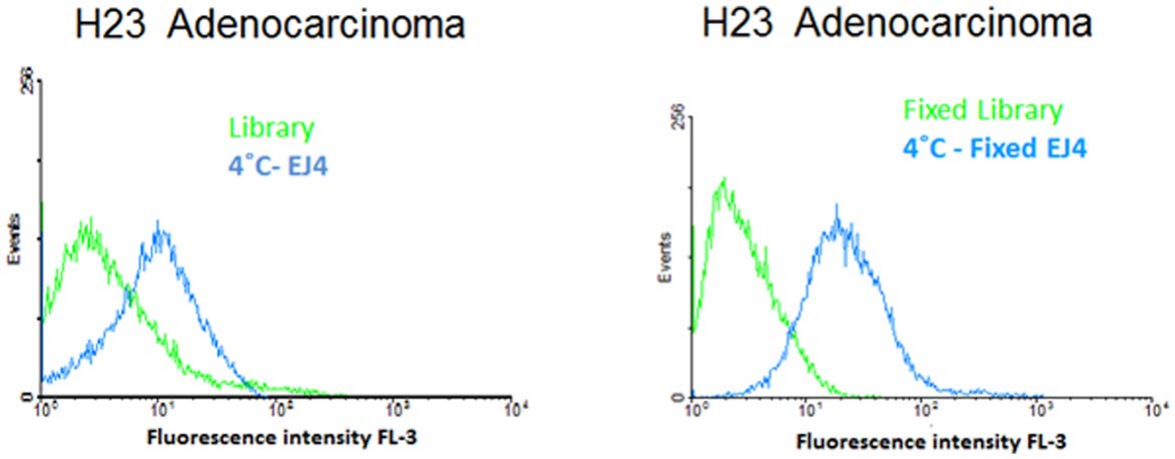
Binding assays after fixation with 10% formalin. Binding assay of aptamers with H23 (target) cells pre-fixed with 10% formalin. Left column shows the binding of selected aptamers with untreated cells at 4°C. Right column shows the binding of selected aptamers fixed cells. The light green curve represents the background binding of a random sequence (library). **Aim figure 4**: The aim of this figure is to show the binding capability of the selected aptamer EJ4 to cells that have been fixed with 10% formalin.

Cancer detection relies on the examination of tissue from biopsies, which are fixed under chemical conditions to preserve the integrity and antigenicity of tumor samples for later use. An important assay in cancer diagnosis is immunostaining, in which freshly dissected tissues are fixed prior to treatment with probes, such as antibodies and aptamers, specifically for tumor markers. Previous studies have shown that aptamers are capable of labeling formalin-fixed paraffin tissue (FFPE) after deparaffinization and antigen retrieval [27]–[28]. To determine the binding capability of the selected aptamers under those conditions, cells were fixed with 10% formalin before incubation with labeled aptamers. As control for these experiments, two known aptamers for leukemia, Sgc8 and TD05, and their corresponding binding cell lines were used to show that the process of fixation does not produce any fluorescence signal. Therefore any fluorescence detected should be an indication of a binding event between the aptamers and its target. As shown in figure S3, both controls retained their initial binding profile, indicating that no artificial increment in fluorescence intensity occurred as a result of the fixing conditions. EJ2, EJ5, EJ7, ADE1, and ADE2 aptamers maintained similar binding to that displayed at 4°C (Figure 4) indicating that aptamers can bind to their targets even under fixed conditions. These results strongly suggest that selected aptamers have the potential to be used as recognition molecules in clinical samples.

**Figure 5:**
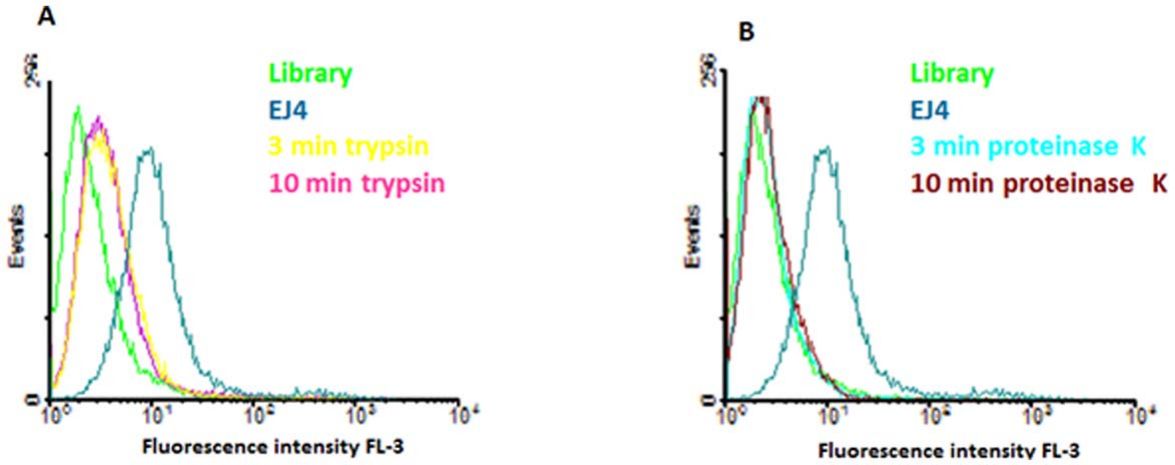
Binding Assays after proteinase treatment. Flow cytometry assay for selected aptamers after treatment with proteases; untreated cells were used as positive control. (A) Cells treated with trypsin for 3 and 10 min prior binding with selected aptamer EJ4. (B) Cells treated with proteinase K for 3 and 10 min prior binding with aptamer EJ4. **Aim figure 5:** The aim of this figure is to prove that the targets of the selected aptamers are proteins present in the cell surface of the cancer cells (H23).

During cell selection, it is expected that aptamers interact specifically with the surfaces of target cells; this behavior has been reported in several studies [29]–[31]. In our experiment, cells were treated with two enzymes, trypsin and proteinase K, for 3 and 10 minutes, washed with PBS, and then incubated with aptamers. All selected aptamers lost binding after treatment with both proteases (Figure 5 and Figure S5. For all experiments, the fluorescence intensity was significantly reduced; however, the signal corresponding to assays with proteinase decreased to the background, indicating that the target protein was removed completely after treatment.

### Conclusion

In conclusion, we have selected a panel of aptamers capable of distinguishing lung carcinoma from normal lung epithelial cells. These aptamers show high affinity towards the H23 cell line with apparent K_d_'_s_ in the nanomolar range, but no detectable affinity for normal lung epithelial cells. Aptamers also showed binding under physiological conditions, as well as after chemical fixation, suggesting that they can be applicable for *in vivo* experiments. Proteinase treatment indicated that all aptamers in this panel bind to proteins on the target cell surface. All these results suggest broad potential applications of selected aptamers due to their specificity for cancerous tissues but not for healthy cells. These aptamers can also be used as molecular probes in clinical samples, such as freshly extracted tumors and preserved histology specimens.

## Materials and Methods

### Library Design

The primers were design to satisfy the following characteristics: a minimum hairpin structure, similar melting temperature (T_m_) and minimal base pairing. The primers and an 80-mer library were designed using the IDT Oligo Analyzer 3.1 software [32]. The forward primer was labeled with Fluorescein Isothiocyanate (FITC) at the 5′-end, and the reverse primer was labeled with Biotin at the 5′-end. The library consisted of a randomized 44-nt region flanked on the 5′-end by the FITC- labeled primer and flanked on the 3′-end by the complementary unlabeled strand of the reverse primer.

### Instrumentation and Reagents

Libraries and primers were synthesized using the 3400 DNA synthesizer (Applied Biosystems). All reagents for DNA synthesis were purchased from Glen Research. DNA sequences were purified by reversed phase HPLC (Varian Prostar using a C18 column and acetonitrile/triethylammonium acetate as the mobile phase). PCR was performed on a Biorad Thermocycler, and all reagents were purchased from Takara. The monitoring of the selection process, binding assays, and determination of the dissociation constants for the selected aptamers were performed by flow cytometric analysis using a FACScan cytometer (BD Immunocytometry Systems).

### Cell Culture and Buffers

A total of eight established cell lines were used in this project, all purchased from the American Tissue Culture Collection (ATCC). H23 (CRL-5800) adenocarcinoma NSCLC was chosen as the positive cell line, while the negative cell line chosen was HBE135-E6/E7 (CRL-2741), normal human bronchial epithelial cells. The H23 cell line was maintained in RPMI-1640 (ATCC) culture medium supplemented with 10% Fetal Bovine Serum (FBS heat-inactivated) and 1% penicillin-streptomycin. The normal bronchial lung cell line was maintained in a Keratin Serum-Free Medium supplemented with 5 ng/mL human recombinant EGF, 0.05 mg/mL bovine pituitary extract (Invitrogen), 0.005 mg/mL insulin, and 500 ng/mL hydrocortisone (Sigma-Aldrich). Cells were incubated at 37°C under 5% CO_2_ atmosphere. Other cell lines used for the selectivity assay were the CAOV3 and TOV21G ovarian cancer cell lines, A549 lung adenocarcinoma, H520 lung squamous cell carcinoma, H460 large cell lung carcinoma, and DLD1 colon adenocarcinoma, and all were maintained according to ATCC specifications. During selection, two buffers were used: Washing Buffer (WB) (glucose 0.45% w/v and MgCl_2_ 5 mM in PBS) and Binding Buffer (BB) (1 mg/mL tRNA and 1 mg/mL BSA in WB). All previous reagents were purchased from Sigma-Aldrich.

### *In vitro* Selection

Because both cell lines used during the selection, adenocarcinoma and normal human bronchial epithelial cells, are adherent cell lines, the selection was performed on cell monolayers. About 20 nmol of the synthesized library was dissolved in 700 µL of binding buffer. Before the process was initiated, the DNA pool was denatured by heating at 95°C for five minutes, followed by rapid cooling on ice. This forced the DNA sequences to adopt the most favorable secondary structures. The DNA library was then incubated with approximately 3×10^6^ target cells (H23) at 4°C for 30 minutes. Subsequently, the cells were washed 3 times with washing buffer to remove unbound sequences. Afterwards, the bound sequences were recovered by heating at 95°C for 10 minutes and then centrifuging at 14,000 rpm to remove cell debris.

The supernatant containing the DNA sequences was collected, and the selected pool was PCR-amplified using FITC- and biotin-labeled primers. Then, the generation of single strand DNA (ssDNA) was achieved by incubation with streptavidin-coated sepharose beads, obtaining the biotinylated strand. Counter selection was carried out after observing some enrichment with the target cells. In order to select aptamers with high specificity and selectivity, the stringency of the washes (number of washes, wash time, and volume of washing buffer) was increased in subsequent rounds. The enrichment of the pools was monitored using flow cytometry, sequenced with 454 technology, and analyzed for aptamer candidates.

### Flow Cytometric Analysis

Flow cytometry was used to monitor the enrichment of ssDNA-bound sequences within the pools during the selection process, as well as to evaluate the binding affinity and specificity of the selected aptamers. The cultured cells were washed with WB before and after incubation with the FITC ssDNA pool or selected DNA sequences. Fluorescence intensity was determined on a FACScan cytometer (BD Immunocytometry).

### 454 Sequencing and Analysis

After 18 rounds of selection, enriched pools 14, 16, and 18 were chosen for sequencing. 454-specific primers and MID were PCR-amplified and added to each sequence contained in each pool, yielding a 125-bp product. The reactions were confirmed by gel electrophoresis, cleaned using a PCR purification kit (Qiagen), and submitted to the ICBR core at the University of Florida for analysis.

Around seven thousand sequences were retrieved and analyzed. First, sequences were grouped on the basis of their MID to determine the corresponding enriched pool. Second, primers and MID were removed by the Perl program in order to leave only the random portion of the sequence for homology analysis with the MAFFT 6.0 program.

### Binding Assays

Different families of sequences retrieved from multiple sequence analyses were synthesized, biotin-labeled at the 3′-end, and tested for binding with the positive and negative cell line. In addition, the binding selectivity of each candidate was determined by incubating a 250 nM aptamer solution with 4×10^5^ target or counter-cells for 30 minutes at 4°C. Cells were washed twice with WB and incubated with streptavidin PE beads for 20 minutes at 4°C. After washing, the cells were suspended in 200 µL WB. The fluorescence was determined with a FACScan cytometer by counting 3×10^4^ events. A randomized 80-mer sequence was used as a control.

### Binding Assays with fixed cells

To determine the ability of the aptamers to bind to fixed cells, procedures similar to those described above were followed, with the exception of the initial treatment of the cells. Briefly, adherent H23 cells were detached from the dish by incubation with non-enzymatic cell dissociation solution, and then fixed with 10% formalin solution for 15 min at 4°C, washed, and suspended in binding buffer.

### Selectivity and Specificity Assays

To determine the specificity of these aptamers, different cell lines, including CAOV3, DLD1, A549, H520, H460, and TOV21G, were used in binding assays. Fluorescence intensity was measured in order to confirm binding. All experiments followed the flow cytometry experimental procedure described above.

### Temperature effect on aptamer binding

Selection was performed at 4°C.To determine if temperature would affect the binding between aptamer and the target cells, binding assays were carried out at two additional temperatures, 25°C and 37°C.

### Determination of the dissociation constant

The affinity of the aptamer and its target was evaluated by saturation assay. A sample containing 5×10^5^ H23 cells was washed and incubated with different concentrations of aptamer until saturation was achieved. All binding assays were repeated three times, and the mean fluorescence intensity was calculated by subtracting the fluorescence intensity of a scrambled sequence. Data were collected, and the dissociation constant (K_d_) was obtained by fitting to a single binding site saturation model using SigmaPlot 11.2v (Jandel, San Rafael, CA).

### Protease Digestion

#### Trypsin and Proteinase K treatment

H23 cells were washed twice with PBS and then incubated with 3 mL of either 0.05% trypsin/0.53 mM EDTA in Hank's balanced salt solution (HBSS), Cellgro, or 0.1 mg/mL proteinase K in PBS at 37°C for 3 and 10 minutes. FBS was added to quench the proteinase activity. Cells were washed with WB and subsequently used for the binding assays described above.

## Supporting Information

Figure S1 **Characterization of selected aptamers.** Flow cytometry assay for the binding of the aptamers ADE1, ADE2, EJ2, EJ5 and EJ7 with H23 (target cell line) and HBE135 E6/E7 (negative cell line). The green curve represents the background binding of a random sequence (library).
(TIF)

Figure S2 **Aptamer binding in physiological conditions.** Flow cytometry assay for the binding of aptamers ADE1, ADE2, EJ4, EJ5 and EJ7 with H23 (target cell line) at 25°C and 37°C. In this set of experiments, binding at 4°C was used as positive control. The green curve represents the background binding of a random sequence (library).
(TIF)

Figure S3 **Control experiment for Fixed treated cells.** Binding assay of aptamer Sgc8 with (A) CEM (target) cells and (B) Ramos (control) cells before (left) and after (right) fixation, showing that no artificial fluorescence signal was produced.
(TIF)

Figure S4 **Binding assays after fixation with 10% formalin.** Binding assay of aptamers with H23 (target) cells pre-fixed with 10% formalin. Left columns show the binding of aptamer ADE1, ADE2, EJ2, EJ5 and EJ7 with untreated cells at 4°C. Right column shows the binding of the same aptamers EJ5 with fixed cells. The light green curve represents the background binding of a random sequence (library).
(TIF)

Figure S5 **Binding Assays after proteinase treatment.** Flow cytometry assay for aptamers ADE1 and ADE2, EJ5 and EJ7 after treatment with proteases; untreated cells were used as positive control. (A) and (C) Cells treated with trypsin for 3 and 10 min prior binding with aptamers ADE1 and ADE2, EJ5, and EJ7 respectively. (C) and (D) Cells treated with proteinase K for 3 and 10 min prior binding with aptamers respectively.
(TIF)

Figure S6 **Apparent Kd's for selected aptamers.** Saturation binding curves for selected aptamers ADE1, ADE2, EJ2, EJ5 and EJ7. Cells were incubated with different concentrations of the aptamer in triplicate. The mean fluorescence intensity of unselected library was subtracted from each corresponding aptamer concentration.
(TIF)
