# Supplementary figures and images for: Generation of Lung Adenocarcinoma DNA Aptamers for Cancer Studies
Source: PLoS One. 2012 Oct 17;7(10):e46222. doi: 10.1371/journal.pone.0046222 (PMC3474832; doi:10.1371/journal.pone.0046222)

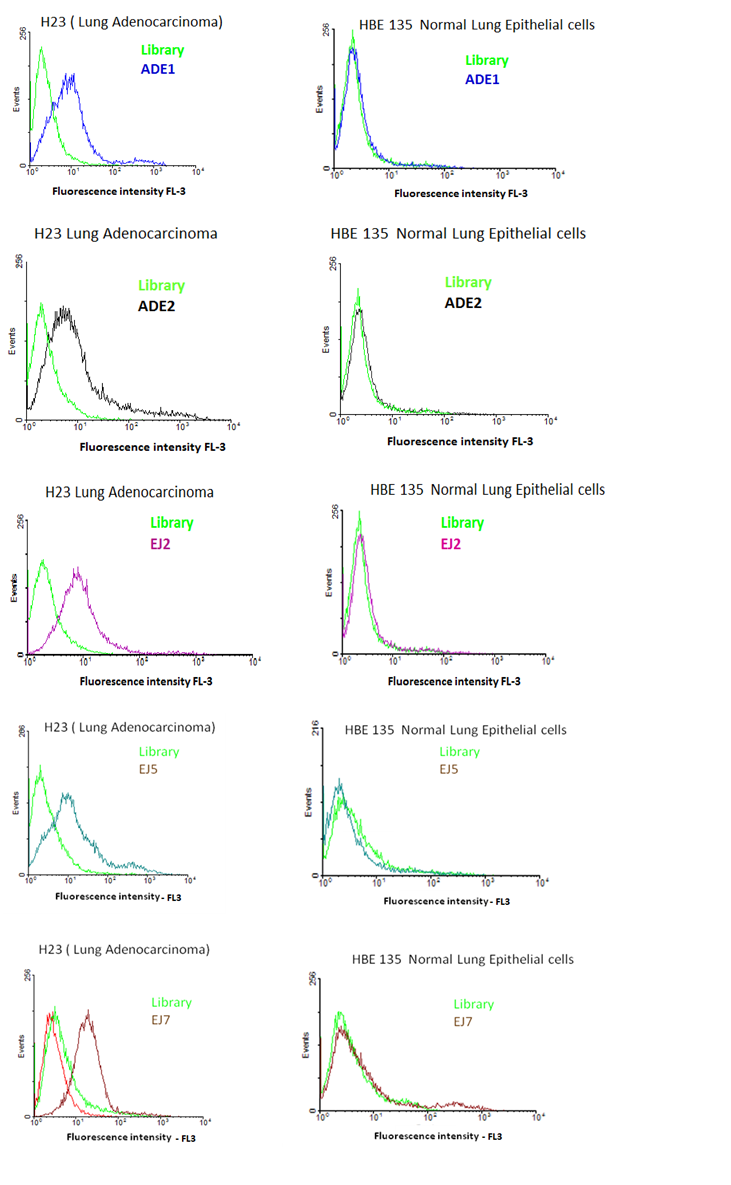

Supplement: Figure S1 — Characterization of selected aptamers. Flow cytometry assay for the binding of the aptamers ADE1, ADE2, EJ2, EJ5 and EJ7 with H23 (target cell line) and HBE135 E6/E7 (negative cell line). The green curve represents the background binding of a random sequence (library). (TIF) [file pone.0046222.s001.tif]

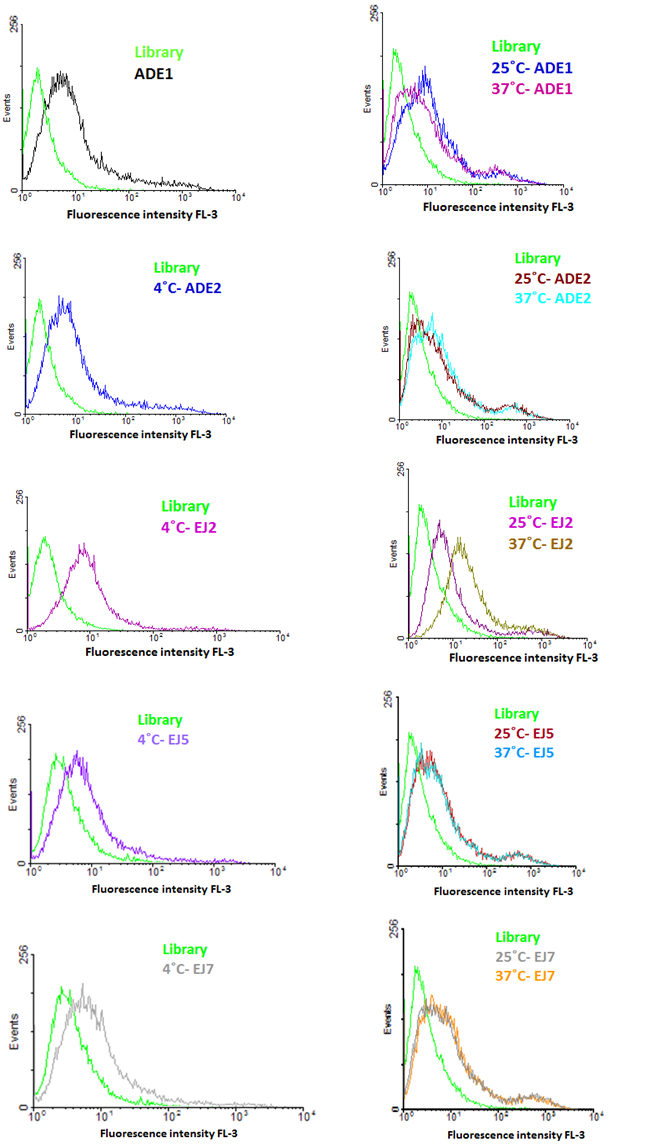

Supplement: Figure S2 — Aptamer binding in physiological conditions. Flow cytometry assay for the binding of aptamers ADE1, ADE2, EJ4, EJ5 and EJ7 with H23 (target cell line) at 25°C and 37°C. In this set of experiments, binding at 4°C was used as positive control. The green curve represents the background binding of a random sequence (library). (TIF) [file pone.0046222.s002.tif]

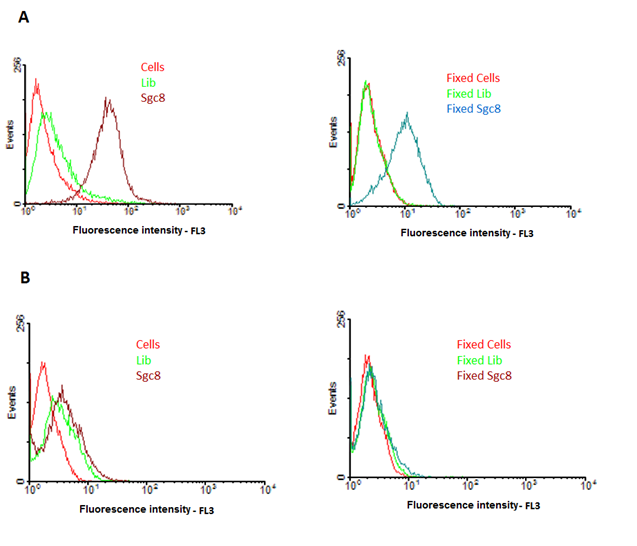

Supplement: Figure S3 — Control experiment for Fixed treated cells. Binding assay of aptamer Sgc8 with (A) CEM (target) cells and (B) Ramos (control) cells before (left) and after (right) fixation, showing that no artificial fluorescence signal was produced. (TIF) [file pone.0046222.s003.tif]

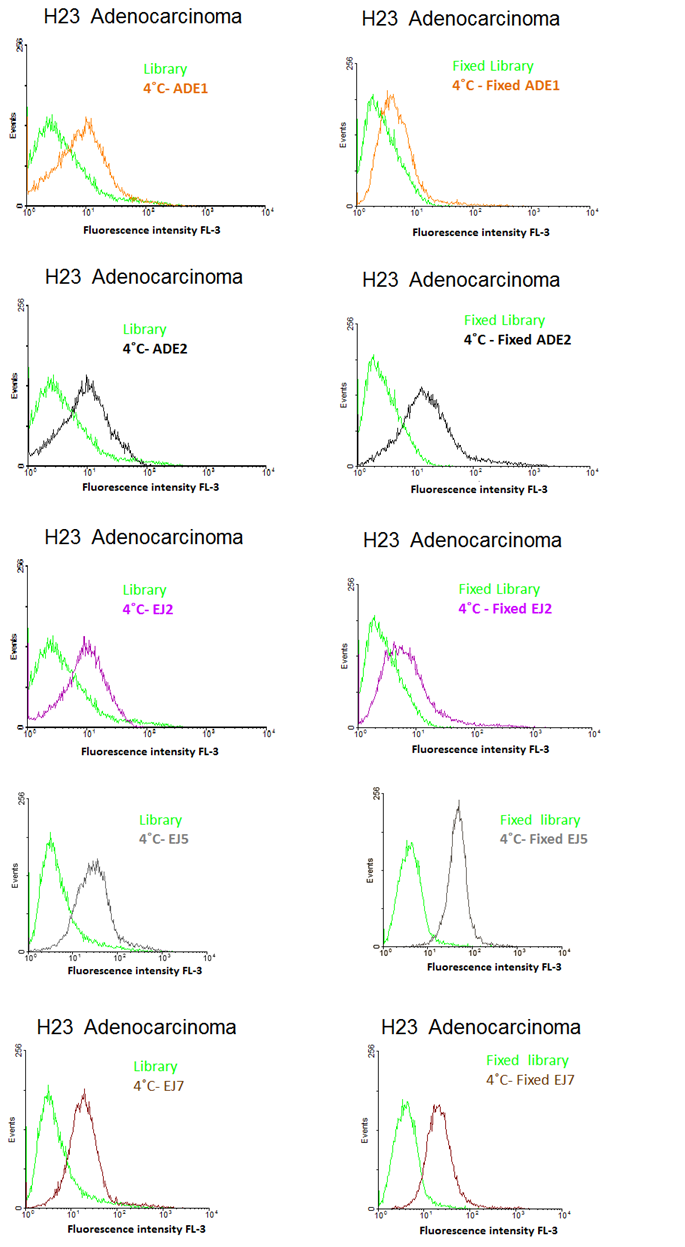

Supplement: Figure S4 — Binding assays after fixation with 10% formalin. Binding assay of aptamers with H23 (target) cells pre-fixed with 10% formalin. Left columns show the binding of aptamer ADE1, ADE2, EJ2, EJ5 and EJ7 with untreated cells at 4°C. Right column shows the binding of the same aptamers EJ5 with fixed cells. The light green curve represents the background binding of a random sequence (library). (TIF) [file pone.0046222.s004.tif]

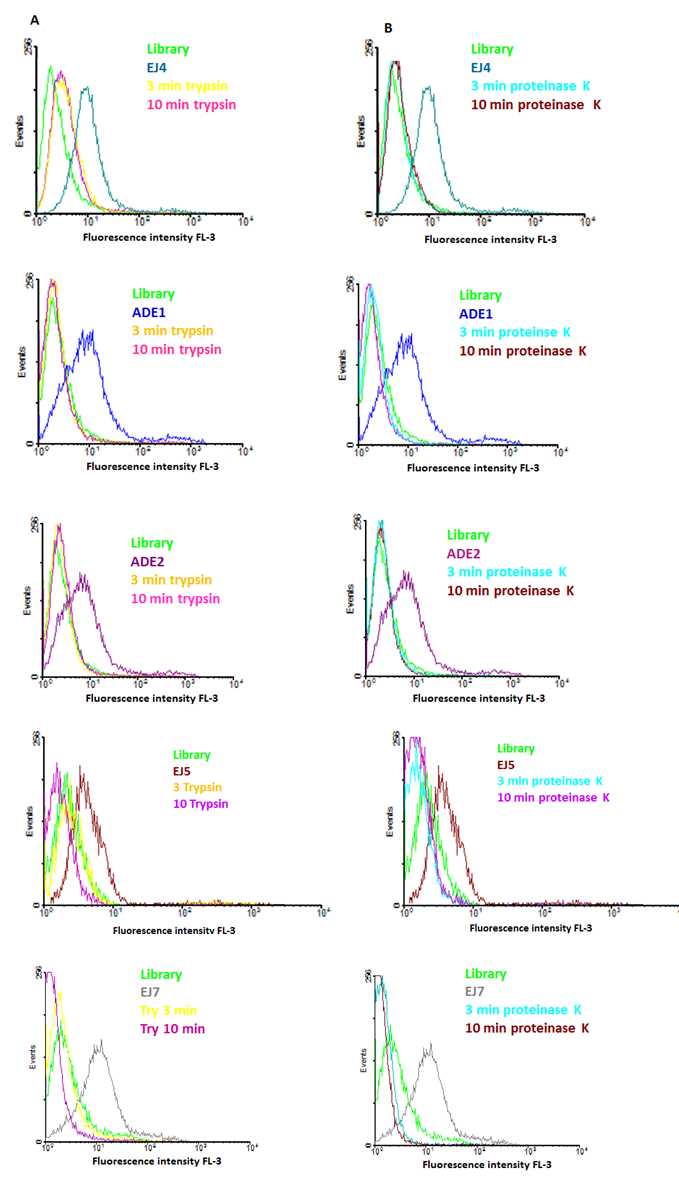

Supplement: Figure S5 — Binding Assays after proteinase treatment. Flow cytometry assay for aptamers ADE1 and ADE2, EJ5 and EJ7 after treatment with proteases; untreated cells were used as positive control. (A) and (C) Cells treated with trypsin for 3 and 10 min prior binding with aptamers ADE1 and ADE2, EJ5, and EJ7 respectively. (C) and (D) Cells treated with proteinase K for 3 and 10 min prior binding with aptamers respectively. (TIF) [file pone.0046222.s005.tif]

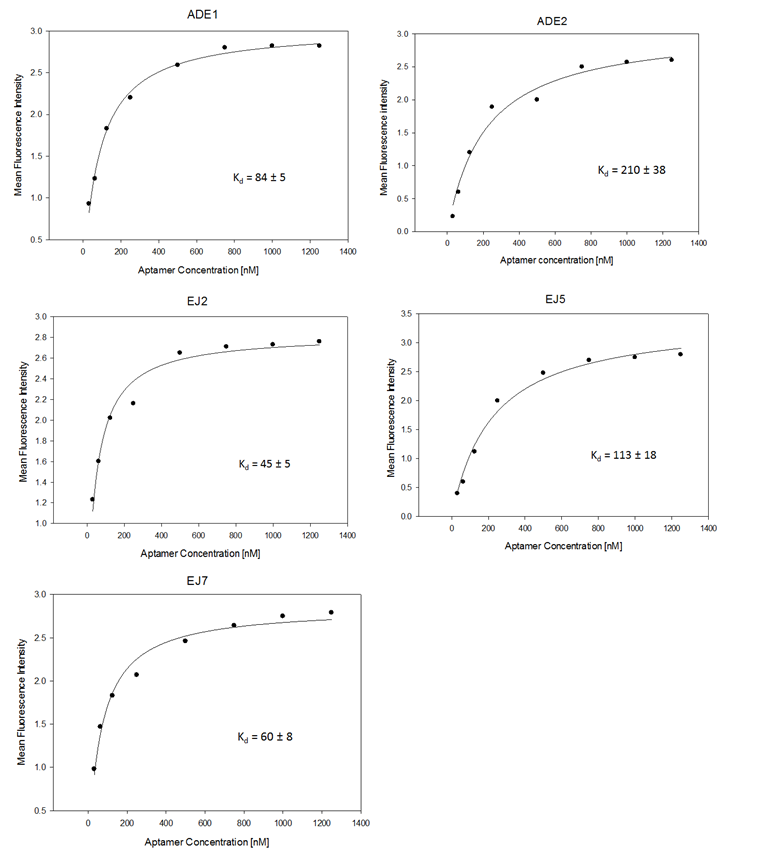

Supplement: Figure S6 — Apparent Kd's for selected aptamers. Saturation binding curves for selected aptamers ADE1, ADE2, EJ2, EJ5 and EJ7. Cells were incubated with different concentrations of the aptamer in triplicate. The mean fluorescence intensity of unselected library was subtracted from each corresponding aptamer concentration. (TIF) [file pone.0046222.s006.tif]
